# The Sexually Antagonistic Genes of *Drosophila melanogaster*

**DOI:** 10.1371/journal.pbio.1000335

**Published:** 2010-03-16

**Authors:** Paolo Innocenti, Edward H. Morrow

**Affiliations:** Department of Animal Ecology, Evolutionary Biology Centre, Uppsala University, Uppsala, Sweden; University of Bath, United Kingdom

## Abstract

When selective pressures differ between males and females, the genes experiencing these conflicting evolutionary forces are said to be sexually antagonistic. Although the phenotypic effect of these genes has been documented in both wild and laboratory populations, their identity, number, and location remains unknown. Here, by combining data on sex-specific fitness and genome-wide transcript abundance in a quantitative genetic framework, we identified a group of candidate genes experiencing sexually antagonistic selection in the adult, which correspond to 8% of *Drosophila melanogaster* genes. As predicted, the X chromosome is enriched for these genes, but surprisingly they represent only a small proportion of the total number of sex-biased transcripts, indicating that the latter is a poor predictor of sexual antagonism. Furthermore, the majority of genes whose expression profiles showed a significant relationship with either male or female adult fitness are also sexually antagonistic. These results provide a first insight into the genetic basis of intralocus sexual conflict and indicate that genetic variation for fitness is dominated and maintained by sexual antagonism, potentially neutralizing any indirect genetic benefits of sexual selection.

## Abstract 2

An association between sex-specific fitness and gene expression in the fruit fly provides an estimate of number, identity and function of sexually antagonistic genes in this species.

## Author Summary

Males and females of many species are different: many of these differences are thought to have evolved because the sexes often have needs and strategies that do not coincide. For example, in fruit-flies, females may do best by concentrating their efforts in acquiring resources to be able to lay more eggs, while males would benefit most from increasing their mating and fertilization success. Such differences generate a sexual “conflict of interests”, and since as a general rule each behavioural, morphological or physiological characteristic is regulated by the same set of genes in the two sexes, this conflict takes place ultimately at the genetic level. In our study, we combined data on the reproductive success of different lines of fruit-flies with their gene expression profiles. We show that a large proportion of genes that contribute to male fertilization success are detrimental for female fecundity, and vice-versa. These results indicate that an optimal genotype for both sexes does not exist: many genes maintain different variants because they have opposite effects in males and females, perhaps helping to explain how genetic diversity is maintained in the face of selection.

## Introduction

Males and females differ in the optimal value for most behavioural, morphological, and physiological traits [1], as a consequence of the different strategies they adopt to maximize their fitness [2],[3]. At the genetic level, these differences trigger an evolutionary conflict between the sexes. For any given genetic locus, an allele may be favoured by selection in males, while a different allele is favoured in females. Hence, intralocus sexual conflict occurs when selection acts differentially on the same locus in the two sexes [4]. If many loci experience this sexually antagonistic selection, sets of alleles that are positively selected in males will produce a “good” male phenotype but a “bad” female phenotype, while the opposite will be true for other sets of alleles positively selected in females. Over the past decade, the phenotypic effects of intralocus sexual conflict have been demonstrated using two major lines of evidence: first, from studies showing a negative genetic correlation for fitness between the sexes, both in wild and laboratory populations [5],[6], and second, from experimental evolution studies, where gender-limited selection resulted in relatively higher fitness of the selected sex [7],[8]. Furthermore, sexually antagonistic selection appears to be a taxonomically widespread phenomenon [9].

Although the effects of intralocus sexual conflict on the whole organism are receiving increasing attention [10], very little is known about the genetics underlying the patterns observed, namely the identity, number, or location of the genes involved. So far, two predictions have been made about the features of sexually antagonistic genes. First, sexually antagonistic loci should accumulate on the sex chromosomes [1] due to their patterns of inheritance in the two sexes [11]. Second, since the genetic information available to males and females is largely coincidental, sexual dimorphism is expected to arise through differences in where, when, and to what extent genes are expressed [12], as a way to resolve the conflict and to mitigate the “gender load” [1]. Numerous studies have employed sex bias in gene expression as a proxy for sexual antagonism [13]–[17] with the assumption that sexual dimorphism in expression levels reflects the current extent to which sexual conflict is present at each locus. However, as some authors explicitly note [9],[12],[13], sex-biased expression is more likely to represent a partial or total resolution to the conflict, and the assumption that sex-biased expression equals sexual antagonism remains to be demonstrated. An explicit test of these predictions at the gene level is only possible when a set of candidate genes has been identified. The aim of this study was therefore to provide an empirical test of current sexual conflict theory with respect to the genome-wide number, location, and function of sexually antagonistic genes in an outbred population of *D. melanogaster*.

## Results/Discussion

**Figure 1:**
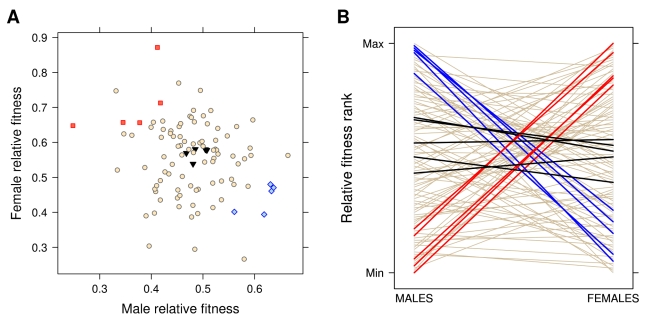
Fitness assay data. (A) Average male and female adult relative fitness (male fertilization success and female fecundity) across 100 hemiclonal lines. (B) Interaction plot of male and female fitness rank for each hemiclone line. In both panels, the 15 selected lines are highlighted in blue (high-male/low-female fitness), red (low-male/high-female fitness), or black (average male and female fitness).

We began by using a quantitative genetic hemiclonal analysis of adult fitness across 100 genomic haplotypes when expressed as either males or females (see Materials and Methods). Adult fitness was measured in terms of fertilization success for males and fecundity for females, both assayed under competitive conditions: these components closely match total adult fitness in our study population [18]. A mixed model was fitted to partition the total phenotypic variance into sex-specific genetic components and their correlation. Consistent with previous studies [6],[11], we found a significant negative genetic correlation for adult fitness between the sexes across these 100 lines (*r*_MF_  = −0.52, 95% Credible Interval: −0.86; −0.10, Table 1). The sex-specific heritabilities were both significantly different from zero, but the estimate for males was much smaller than for females, as previously shown in different species [19],[20],[21]. This sexually antagonistic variation for fitness is illustrated by the negative relationship between male and female relative fitness (Table 1 and Figure 1A) and the crossing pattern in an interaction plot (Figure 1B), demonstrating that genomes with high fitness when expressed in males typically produce low fitness females and vice versa [6].

**Table 1:** Heritability and intersexual genetic correlation for adult fitness.

	Var. comp.	95% C.I.	*h* ^2^	95% C.I.	CV
Female	0.0070	0.0042; 0.0107	0.632	0.428; 0.859	21.28
Female residual	0.0153				31.49
Male	0.0014	0.0005; 0.0030	0.115	0.037; 0.245	11.12
Male residual	0.0222				43.96

	Covariance	95% C.I.	*r*_MF_	95% C.I.	
Male - Female	−0.0016	−0.0033; −0.0001	−0.523	−0.860; −0.103	

A mixed model was used to partition the phenotypic variance for male and female adult fitness and to estimate the intersexual genetic correlation. Abbreviations: Var. Comp., variance component; *h^2^*, narrow sense heritability; *CV*, coefficient of variation for the sex-specific additive genetic components (CV_A_) and for the residual variances (CV_R_); *r_MF_*, intersexual genetic correlation.

**Figure 2:**
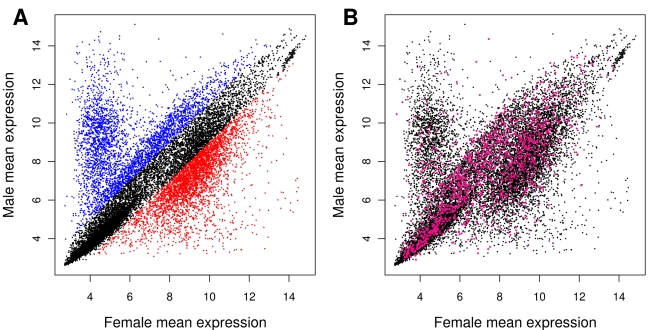
Gene expression data. Mean expression values in males and females for each transcript. (A) Male-biased and female-biased transcripts showing 2-fold or greater differences in gene expression are represented with blue and red dots, respectively. (B) Purple dots represent transcripts showing significant interaction between sex and fitness in the regression on gene expression.

After establishing the presence of sexually antagonistic variation for fitness, we undertook a gene expression analysis on a subset of the original 100 lines. We selected five lines for which fitness was high in males but low in females, five lines showing the opposite pattern, and five lines showing average fitness across both sexes (Figure 1B). Gene expression was measured in males and females of the selected lines during the peak of their reproductive activity using Affymetrix Drosophila GeneChip 2.0 microarrays. For each transcript, we fitted a mixed model to partition the variance in expression between sexes, among lines, and their interaction. An additional factor was introduced to control for the batch effect in microarray hybridisation. The effect of sex was significant for 17,350 transcripts (91.5% of the transcriptome) at a false discovery rate (FDR) of 0.001, indicating extensive sex-biased gene expression. When the magnitude of differential expression was considered, 7,490 of the significant transcripts showed greater than 2-fold change, 3,652 showing male-biased expression and 3,838 female-biased expression (Figure 2A) [22]. Genetic variation in gene expression (the line term) was significant for 5,173 transcripts (27.3%, FDR <0.001), while the interaction term was significant for 2,151 transcripts (11.3%, FDR <0.001). This latter effect represents the amount of genetic variation for sexual dimorphism, the prerequisite for the independent evolution of the sexes towards their respective fitness optima [23]. While these data are consistent with a pattern of sexual antagonism in the genome, they are not sufficient in themselves to establish which genes are currently experiencing sexual conflict. In order to identify those candidate loci, we used a regression model to test the association between gene expression and sex, fitness and their interaction: the expression level of the sexually antagonistic loci will be associated with a significant interaction between sex and fitness. Before testing this full model, we began by fitting two regression models to male and female data separately, to later establish what proportion of transcripts associated with sex-specific fitness are also sexually antagonistic.

**Figure 3:**
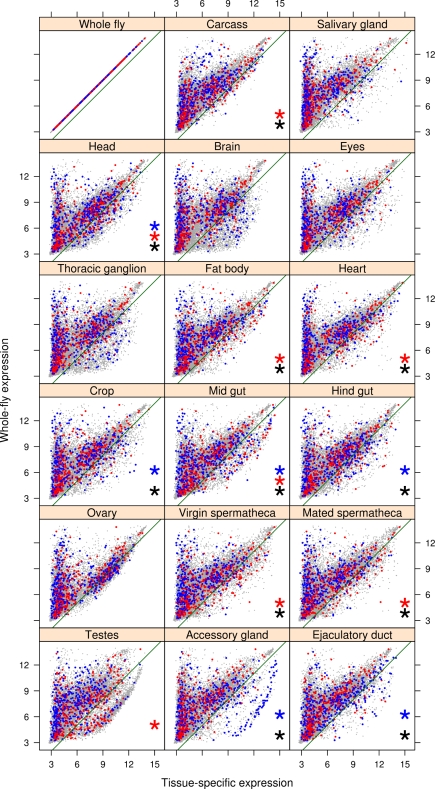
Tissue-specific expression of genes associated with male fitness. Expression levels of transcripts in different tissues (*x*-axis) against the expression levels in the whole fly (*y*-axis); data from FlyAtlas [38]. The green line represents the cut-off below which the transcripts are considered tissue-specific. Blue and red dots represent the transcripts positively and negatively associated with male fitness, respectively. Black, blue, and red asterisks represent tissues significantly enriched (adjusted *p* value <0.01) for tissue-specific transcripts associated with male fitness (black, overall; blue, positively associated; red, negatively associated).

In males, 867 transcripts (4.6%, FDR <0.05; see Table S1) were significantly associated with adult fitness, 460 showed a positive association and 407 showed a negative association. By comparing the expression level of these transcripts in the whole fly to their expression in specific tissues using the FlyAtlas database (see Materials and Methods), we were able to determine which tissues were enriched for male fitness-associated genes. Out of the 17 tissues tested (Table S2), we found 11 to be enriched for such genes (Figure 3). Interestingly, the tissues exhibiting the strongest pattern of enrichment for male fitness-associated genes were the accessory gland and ejaculatory duct, both significantly enriched for genes positively associated with male fitness (Fisher's exact test odds ratios 5.16 and 3.75, respectively; see Table S2). These genes showed over-representation in Gene Ontology (GO) categories specifically related to male fertilization success (e.g., insemination, sperm displacement, post-mating behaviour; see Table S3), confirming a large body of literature that has implicated post-mating sexual selection as an important selective force determining adult male reproductive success [24],[25]. Overall, the wide number of tissues and biological processes involved implies that fitness in the adult male fly is a highly complex trait, although post-mating sexual selection appears to be the major selective force operating on males. This pattern is to be expected given that other selective pressures might be reduced in the controlled laboratory environment to which our population has adapted.

**Figure 4:**
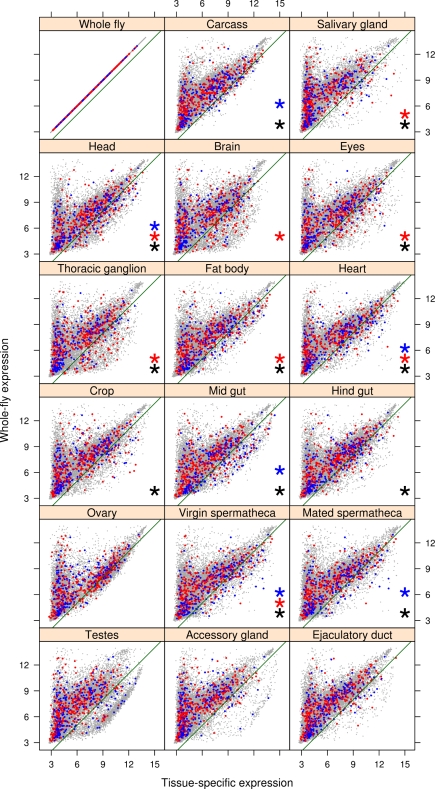
Tissue-specific expression of genes associated with female fitness. Expression levels of transcripts in different tissues (*x*-axis) against the expression levels in the whole fly (*y*-axis); data from FlyAtlas [38]. The green line represents the cut-off below which the transcripts are considered tissue-specific. Blue and red dots represent the transcripts positively and negatively associated with female fitness, respectively. Black, blue, and red asterisks represent tissues significantly enriched (adjusted *p* value <0.01) for tissue-specific transcripts associated with female fitness (black, overall; blue, positively associated; red, negatively associated).

In females, 634 transcripts (3.3%, FDR <0.05; see Table S1) were found to be significantly associated with adult fitness, of which 267 showed a positive association and 367 showed a negative association. The pattern of tissue specificity of these genes in females again involved most tissues (Figure 4 and Table S2) with diverse functions and enriched to a similar extent, making a general interpretation difficult. However, three broad categories were represented: (a) sex-limited tissues involved in reproduction, specifically in storing sperm after mating, the spermatheca (in both virgin and mated female adults) seems to confirm a role of post-mating sexual selection; (b) tissues with a role in metabolism, transport, and storage of nutrients (crop, midgut, hindgut, fat body, and heart); and (c) neural tissues (head and thoracic ganglion). Remarkably, candidate genes expressed in several tissues (carcass, head, fat body, heart, eyes) were enriched for GO categories connected to an immune response or a response to an external toxic stimulus (e.g., defence response, response to xenobiotic stimulus, response to bacteria, insecticide metabolic process; see Table S4), which were absent in males. This is a particularly tantalizing result given the evidence that a post-mating immune response by females is induced by components of male ejaculates [26],[27], suggesting a link between immune system function and fecundity in females.

**Figure 5:**
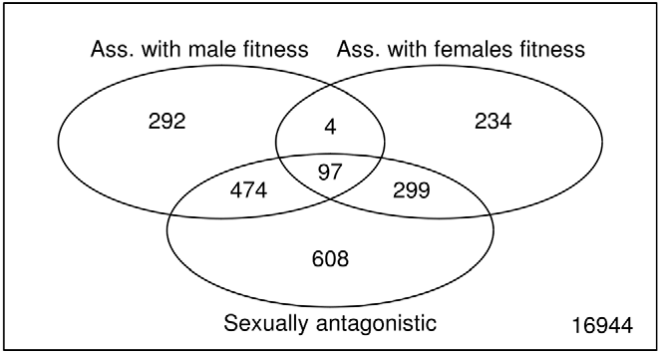
Venn diagram. Intersections between genes significantly (positively or negatively) associated with male fitness, female fitness (from the sex-specific models), and those that show a significant interaction between sex and fitness in the full model (i.e., sexually antagonistic).

When the whole dataset was considered, 608 transcripts (3.2%, FDR <0.05; see Table S1) were associated with fitness, while the sex by fitness interaction term—defining putative sexually antagonistic loci—was associated with 1,478 transcripts (7.8%, FDR <0.05, corresponding to 1,292 known genes; see Figure 2B and Table S1), 817 being male-benefit/female-detriment and 661 being female-benefit/male-detriment (89% and 95% show opposite sign in the regression slope of sex-specific fitness, respectively). The majority of genes associated with sex-specific fitness are also sexually antagonistic (66% and 62% for males and females, respectively; see Figure 5), corroborating the hypothesis that genetic variation for fitness is maintained by sexually antagonistic selection [5]. However, surprisingly these sexually antagonistic loci represent only 8.5% of the total number of sex-biased transcripts. The conspicuous discrepancy between the size and the distribution of these two sets of genes (compare Figure 1A and Figure 1B) suggests that sex-biased expression represents a footprint of widespread but resolved conflict between the sexes, rather than a signature of ongoing antagonism.

**Figure 6:**
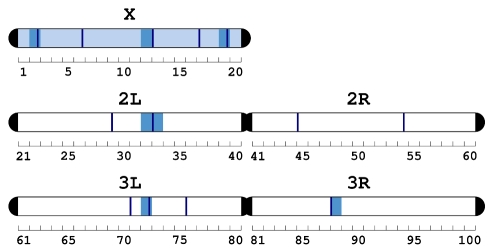
Chromosomal distribution of sexually antagonistic candidate genes. Chromosomes, chromosomal bands (1–100), and sub-bands (A–F in each band, not labelled but qualitatively indicated by their relative position in each band), enriched for sexually antagonistic candidate genes are coloured in light blue, blue, and dark blue, respectively. See Table S5 for details and statistics.

**Figure 7:**
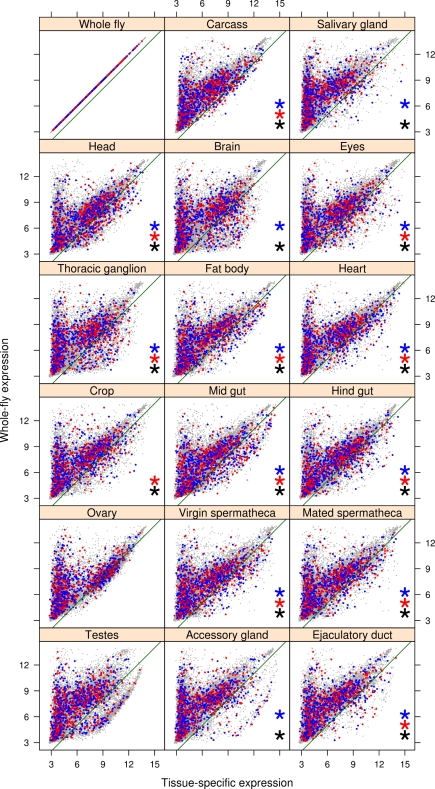
Tissue-specific expression of sexually antagonistic candidate genes. Expression levels of transcripts in different tissues (*x*-axis) against the expression levels in the whole fly (*y*-axis); data from FlyAtlas [38]. The green line represents the cut-off below which the transcripts are considered tissue-specific. Blue and red dots represent the male-beneficial and female-beneficial transcripts, respectively. Black, blue, and red asterisks represent tissues significantly enriched (adjusted *p* value <0.01) for tissue-specific antagonistic genes (black, overall; blue, male-beneficial; red, female-beneficial).

The identification of a list of candidate sexually antagonistic loci enables us to ask where they are located in the genome and which biological processes contribute to the negative genetic correlation for adult fitness, which generates the “gender load” [6]. As many as 68 genomic regions were enriched for sexually antagonistic loci, notably including the X chromosome (odds ratio = 1.16, *p* = 0.029; Figure 6 and Table S5), in line with current theory [1]. All the tissues tested showed enrichment for these candidate genes, with the intriguing exception of the gonads, both testes and ovaries (Figure 7). We may speculate that the paucity of sexually antagonistic genes in the gonads may derive from the highly specific regulatory mechanisms present in the testes and ovaries. The testes in particular have an exceptionally low correlation in gene expression with other tissues (see Figure S3). Thus, the opportunity for sexually antagonistic selection to operate in the gonads may be low. On the other hand, other tissues that are present in only one sex show a statistically significant overabundance of sexually antagonistic genes (accessory gland, ejaculatory duct, spermatheca in both virgin and mated females). Although counterintuitive, this pattern can arise because each gene may either have different functions in both male and female sex-limited organs or show high levels of expression in other shared tissues, where conflict can occur. To graphically assess the plausibility of these hypotheses, we plotted the candidate antagonistic genes that show high tissue specificity for both male-limited (accessory gland and ejaculatory duct; Figure S1) and female-limited tissues (spermatheca; Figure S2) in every other tissue in the adult fly. The resulting patterns support both scenarios: antagonistic genes in male-limited tissues are also, for the vast majority, expressed in the spermatheca, while antagonistic genes in the spermatheca show extremely high correlation in expression with other tissues, such as fat body and heart (Figure S2). In general, the candidate genes we identified are highly expressed in most tissues, and although we ignore whether, as a rule, these genes code for the same function when expressed in different physical locations, these results seem to indicate that pleiotropy can be a mechanism that hampers the resolution of the conflict [16].

Enriched biological processes of genes identified as sexually antagonistic showed similarities to those associated with male and female fitness, with the general pattern emerging of sexual antagonistic selection influencing many diverse processes (Table S6). Taken together, the patterns of sexually antagonistic genes present in almost all tissues influencing genes involved in the regulation of many biological processes suggests that sexual antagonism is a pervasive selective force currently influencing the *D. melanogaster* genome.

That said, it should be noted that our list of candidate sexually antagonistic genes is far from conclusive, for two main reasons. First, we are probably underestimating the intensity of sexual conflict, because our analysis of the fly transcriptome is limited to a narrow window of time in the lifespan of this organism. Although adult, sexually mature flies probably best express the potential for sexual conflict at the transcriptional level, we argue that at other life stages, in particular during development and metamorphosis, alleles at other loci could act antagonistically and contribute to variation in reproductive success. Second, our analysis is based on a laboratory population, where some sources of viability selection—which are less likely to act antagonistically in the two sexes [28]—may be eliminated, potentially exacerbating the relative importance of sexual antagonism. Whether the patterns found in our study can be extrapolated to wild populations remains to be tested.

### Conclusion

Our results provide the first direct test, to our knowledge, of the identity, quantity, and location of sexually antagonistic genes in any organism. These data show that sexually antagonistic selection has a non-negligible effect on fitness-related genes, and as such its neutralizing effect on “good genes” processes in sexual selection should no longer be overlooked [19]. They also give an indication of the extent to which this process may maintain genetic variation in the face of sexual (i.e., the lek paradox [29]) or natural selection [5],[30]. The presence of sexual antagonism in sex-limited tissues other than the gonads also provides evidence of a link between intralocus and interlocus sexual conflict, since the accessory gland in males and sperm-storage organs in females are known to play an important role in male-female coevolution [31],[32]. We expect our results will be a starting point from which a more detailed functional genomic analysis of sexual conflict can proceed. In particular, a better understanding of the function, genomic location, and the degree of linkage in a gene network (epistasis and pleiotropy) of each locus under conflict might provide insights into the processes that allow or prevent conflict resolution [10].

## Materials and Methods

### Stocks and Experimental Methods

The base population of *Drosophila melanogaster* (LH_M_) has been maintained as a large, outbred population for over 400 non-overlapping generations. One hundred haplotypes were sampled from LH_M_ and maintained as heterozygous stock hemiclonal lines using double-X clone-generator females [C(1)DX, *y*, *f*; T(2;3) *rdgC st in ri p* ^P^ *bw* ^D^][6],[18]. Hemiclonal haplotypes were expressed as males by mating stock hemiclone males with virgin double-X LH_M_ females [C(1)DX, *y*, *f*] and expressed as females by mating with virgin LH_M_ females. Each hemiclonal fly therefore shares one nearly complete genomic haplotype (with the exception of the fourth dot chromosome), the other being a random sample from the base population. Given the patterns of inheritance of a hemiclonal genotype, the variation across lines does not include any non-additive dominance variation or maternal effects, although some epistatic interactions remain [18]. Adult fitness of hemiclones was assayed in competition with individuals from a replica population of LH_M_ marked with the *bw* ^−^ eye-colour allele.

### Fitness Assays

All flies were reared in 25 mm vials on cornmeal-molasses-agar food. The total adult lifetime fitness of 100 hemiclonal haplotypes when expressed as either males or females was assayed under competitive conditions that closely match those experienced by adults in the base population [18]. Competitor flies homozygous for the brown eye-colour allele *bw* ^-^ were generated following nine rounds of backcrossing into LH_M_. For the male assays, hemiclonal males were first generated by mating stock hemiclonal males to 30 virgin double-X LH_M_ [C(1)DX, *y*, *f*] females. These females were allowed to oviposit in vials for 18 h, after which the density of eggs was reduced so that approximately 150 viable zygotes remain (3/4 of the zygotes are lethal aneuploids). Five hemiclonal wild-type males arising from this cross were then placed together with 10 competitor *bw* ^−^ males and 15 virgin *bw* ^−^ females (reared at the same larval density and matched for age) in yeasted vials for 2 d. The females were then isolated in test tubes and allowed to oviposit for 18 h. On Day 12, the progeny from each female was scored for eye colour. This assay was replicated 6 times, representing a total of 30 hemiclonal males per line. The relative adult male fitness for each line was calculated by averaging the relative fitness across replicates, obtained by dividing the proportion of offspring sired by hemiclonal males (*bw* ^+^/*bw* ^−^) by the maximum proportion across all hemiclonal lines and replicates. For the female assays, the protocol was identical except that hemiclonal females were obtained by mating hemiclonal stock males to groups of 16 virgin LH_M_ females (producing half aneuploids). Groups of 5 hemiclonal females were housed with 10 competitor females and 15 *bw* ^−^ males in yeasted vials for 2 d. The hemiclonal females were then placed in individual test tubes and allowed to oviposit for 18 h. This assay was replicated 4 times, representing a total of 20 hemiclonal females per line. Relative adult female fitness for each line was calculated by averaging across replicates the mean number of progeny emerging by Day 12 divided by the maximum fecundity across all lines and replicates.

### Fitness Data Analysis

All statistical analyses were performed using R [33] 2.9 (http://www.R-project.org). Fitness assay data were analysed by fitting a linear mixed model using Bayesian methods and Markov chain Monte Carlo sampling techniques (MCMCglmm package) to data on relative male and female fitness: Y  =  S + L + ε, where S (sex) is a fixed effect, L (line) is a 2×2 matrix that specifies the variance structure of the random effects, allowing for estimates of sex-specific variances among lines and their covariance, and ε is a matrix of sex-specific, within-line residual variances. Flat priors for the correlation were used.

### Selection of Lines for Expression Analysis

Fifteen lines showing hyper-dispersed variation in relative male and female fitness based on ranks were selected for expression analysis with DNA microarrays. We chose five lines each showing low-male/high-female fitness ranks, high-male/low-female fitness ranks, and average-male/average-female fitness ranks (see Figure 1) as well as low variance.

### Biological Material for Expression Analysis

Four independent replicates of hemiclonal males and females from each of the 15 selected lines were generated following the same crosses described above (but with 12 hemiclonal stock males:30 females). Adult hemiclonal and LH_M_ tester flies of both sexes (reared following the base population protocol) were then collected in groups of 16 on Day 10. On Day 12, each group of hemiclones was placed together with a group of tester flies of the opposite sex in yeasted vials. After 24 h, the tester flies were removed and after a further 20 h a group of six hemiclonal flies were randomly chosen from each vial under brief CO_2_ anaesthesia. Four hours after sorting, the flies were frozen using liquid nitrogen and stored at −80°C for no more than 6 d until RNA extraction.

### RNA Extractions and Microarrays

Total RNA was extracted using Trizol (Invitrogen) and purified with an RNeasy Mini Kit (Qiagen), from four independent groups of six flies for each sex/line (2 sexes, 15 lines, 4 replicates, giving a total of 120 arrays and 720 flies). RNA quantity and quality was assessed with an Agilent Bioanalyzer (Agilent Technologies) prior to sample preparation and hybridisation following the manufacturer's instructions to GeneChip Drosophila Genome 2.0 Affymetrix microarrays at the Uppsala Array Platform. The 120 microarrays were processed in 8 batches of 15.

### Gene Expression Data Analysis

Several packages within BioConductor [34] 2.4 (http://www.bioconductor.org) were used for gene expression data analysis. Microarray data were pre-processed using Robust Multichip Average (RMA) as implemented by the affy package [35].

The phenotypic variation in gene expression was partitioned using the following linear restricted maximum-likelihood mixed model (lme4 package): Y  =  B + S + L + S × L + ε, where S (sex) is a fixed effect, L (line) is a random effect, and B is a random effect introduced to block for the effect of batch. A similar model (without the S and interaction terms) was fitted to sex-specific subsets of the data. The *p* values for random effects were calculated using a 0.5χ_0_^2^+0.5χ_1_^2^ mixture distribution from a Likelihood Ratio Test on the full and reduced (without the random effect to be evaluated) models. All the reported *p* values were corrected for FDR [36].

We used the following regression model: Y  =  B + S + F + S × F + ε (S  =  sex as fixed effect; F  =  sex-specific line fitness, covariate; B  =  batch as random blocking factor) to identify transcript associated with fitness (limma package). A similar model (without the S and interaction term) was fitted to sex-specific subsets of the data. A Bayesian approach to pool information across genes has been used to moderate the variance [37]. All the reported *p* values were corrected for FDR [36].

We identified tissue-specific transcripts using the Flyatlas database [38]. Raw data were downloaded by GEO (Gene Expression Omnibus, accession number GSE7763) and pre-processed with RMA (as default in affy package [35]) separately for each tissue. Expression values were then averaged across replicates and rescaled to whole-fly baseline expression (also obtained from FlyAtlas, to ensure homogeneity of the experimental procedures) using the average expression of unexpressed genes (*n* = 599, expression value in the whole fly smaller than 3.4). Rescaling was necessary only to ensure an equal signal baseline for all the tissues. Transcripts were considered tissue specific if the expression level in the target tissue was 2-fold higher than in the whole fly. To test for overabundance of genes of interest in a target tissue, we performed a one-tailed Fisher's exact test on the observed and expected tissue-specific genes of interest compared to the overall number of tissues-specific genes in each tissue. All the reported *p* values were Bonferroni-corrected for testing on multiple tissues (*n* = 17).

To identify GO categories and chromosomes (or chromosomal bands) enriched for particular subsets of transcripts, we used a hypergeometric test for overrepresentation (*p* <0.05, GOstats and Category packages, modified).

Microarray data are deposited on the GEO database, accession number GSE17013.

## Supporting Information

Figure S1 **Expression levels of antagonistic genes highly expressed in male-limited tissues.** Antagonistic genes that have high tissue-specific expression in the accessory glands and in the ejaculatory duct are highlighted in green in each panel.
(1.82 MB TIF)

Figure S2 **Expression levels of antagonistic genes highly expressed in female-limited tissues.** Antagonistic genes that have high tissue-specific expression in the spermatheca (mated or virgin) are highlighted in green in each panel.
(1.91 MB TIF)

Figure S3 **Correlation for gene expression in different tissues.** Data from FlyAtlas [38].
(2.04 MB TIF)

Table S1 **Annotation of genes associated with adult fitness.** Affymetrix probeset and gene annotation for each of the transcripts associated with male fitness (Sheet 1), female fitness (Sheet 2), both male and female fitness (Sheet 3), and sexually antagonistic genes (Sheet 4).
(0.66 MB XLS)

Table S2 **Statistics of the Fisher's exact test on tissue-specific expression.** For each tissue (rows) and each list of genes of interest (columns), three values are given: effects size (odds ratio), *p* value, and Bonferroni-corrected *p* value. Nine lists of genes were tested: genes associated with male fitness (“m”, all genes; “m.pos”, positively associated; “m.neg”, negatively associated), genes associated with female fitness (“f”, all genes; “f.pos”, positively associated; “f.neg”, negatively associated), and antagonistic genes (“antag”, all genes; “antag.mplus”, male beneficial; “antag.fplus”, female beneficial).
(0.02 MB XLS)

Table S3 **Gene Ontology categories enriched for genes associated with male fitness.** Subsets of fitness-related transcripts showing tissue-specific expression were tested for overrepresentation of GO terms in each tissue.
(0.03 MB XLS)

Table S4 **Gene Ontology categories enriched for genes associated with female fitness.** Subsets of fitness-related transcripts showing tissue-specific expression were tested for overrepresentation of GO terms in each tissue.
(0.06 MB XLS)

Table S5 **Chromosomal distribution of sexually antagonistic genes.** Chromosomes, chromosomal bands, and sub-bands enriched for sexually antagonistic genes.
(0.01 MB XLS)

Table S6 **Gene Ontology categories enriched for sexually antagonistic genes.** Subsets of fitness-related transcripts showing tissue-specific expression were tested for overrepresentation of GO terms in each tissue.
(0.09 MB XLS)

## Abbreviations

FDR: false discovery rate
GEO: Gene Expression Omnibus
GO: Gene Ontology
RMA: Robust Multichip Average
