# Supplementary figures and images for: The Sexually Antagonistic Genes of Drosophila melanogaster
Source: PLoS Biol. 2010 Mar 16;8(3):e1000335. doi: 10.1371/journal.pbio.1000335 (PMC2838750; doi:10.1371/journal.pbio.1000335)

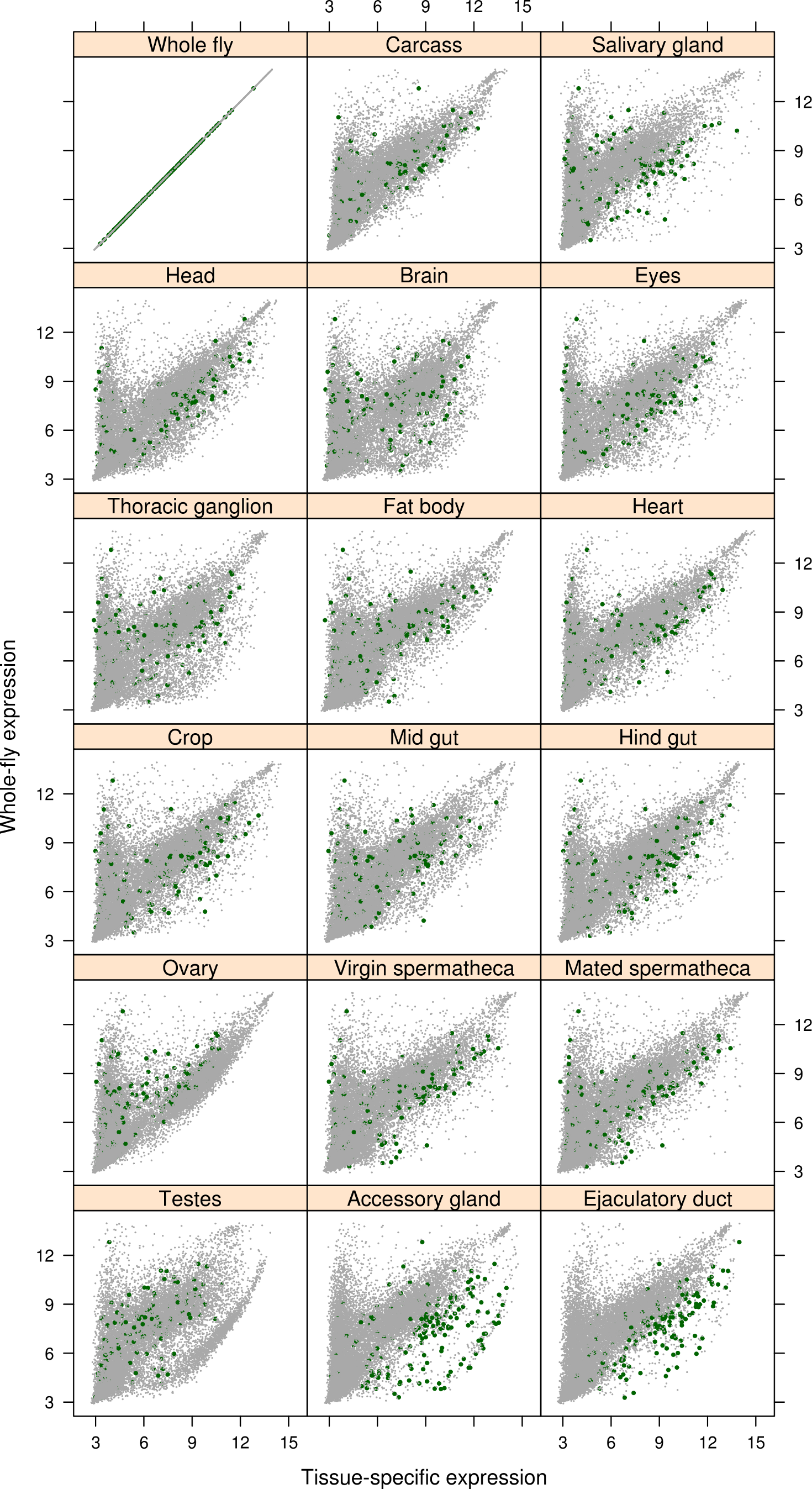

Supplement: Figure S1 — Expression levels of antagonistic genes highly expressed in male-limited tissues. Antagonistic genes that have high tissue-specific expression in the accessory glands and in the ejaculatory duct are highlighted in green in each panel. (1.82 MB TIF) [file pbio.1000335.s001.tif]

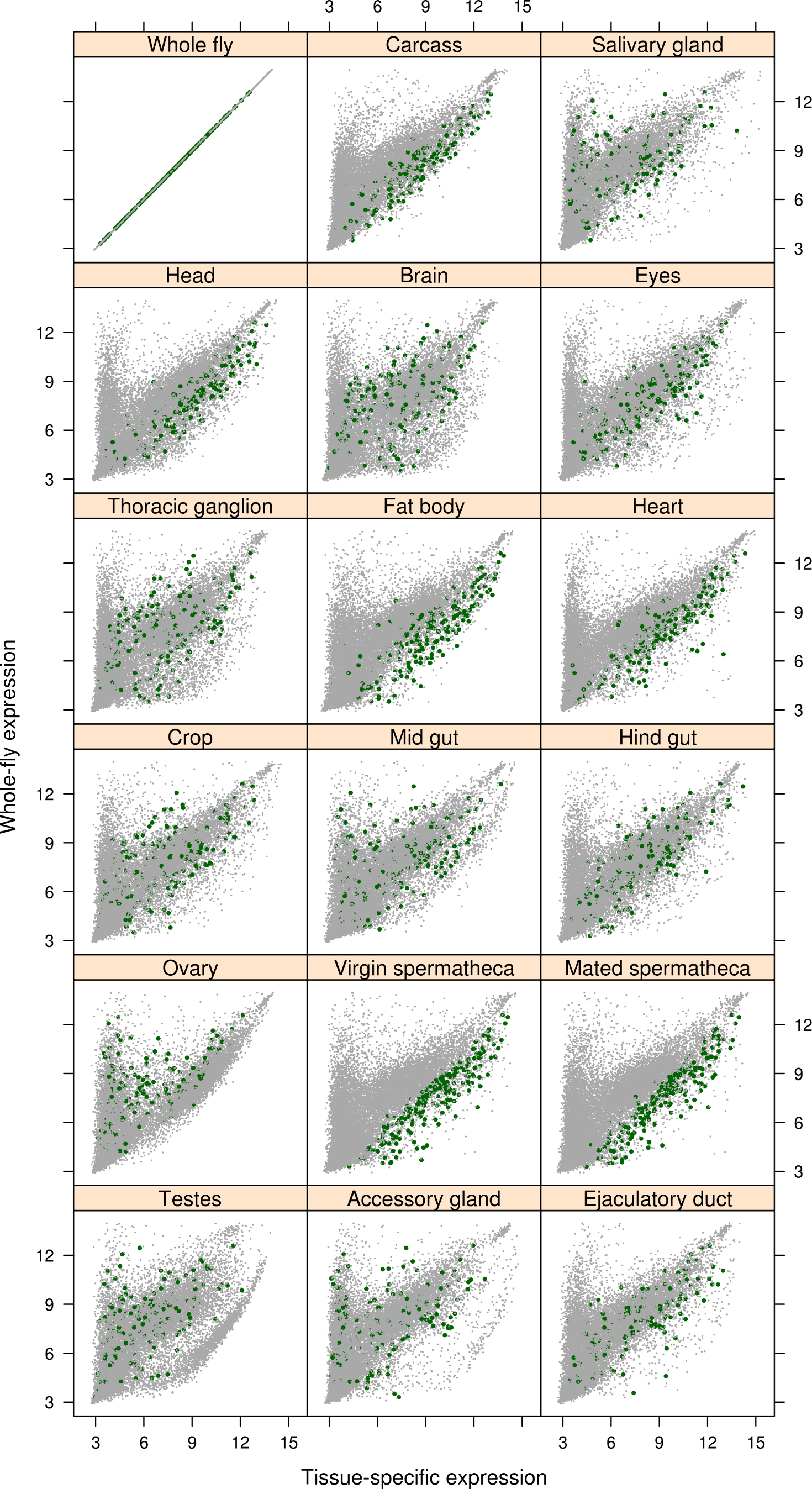

Supplement: Figure S2 — Expression levels of antagonistic genes highly expressed in female-limited tissues. Antagonistic genes that have high tissue-specific expression in the spermatheca (mated or virgin) are highlighted in green in each panel. (1.91 MB TIF) [file pbio.1000335.s002.tif]

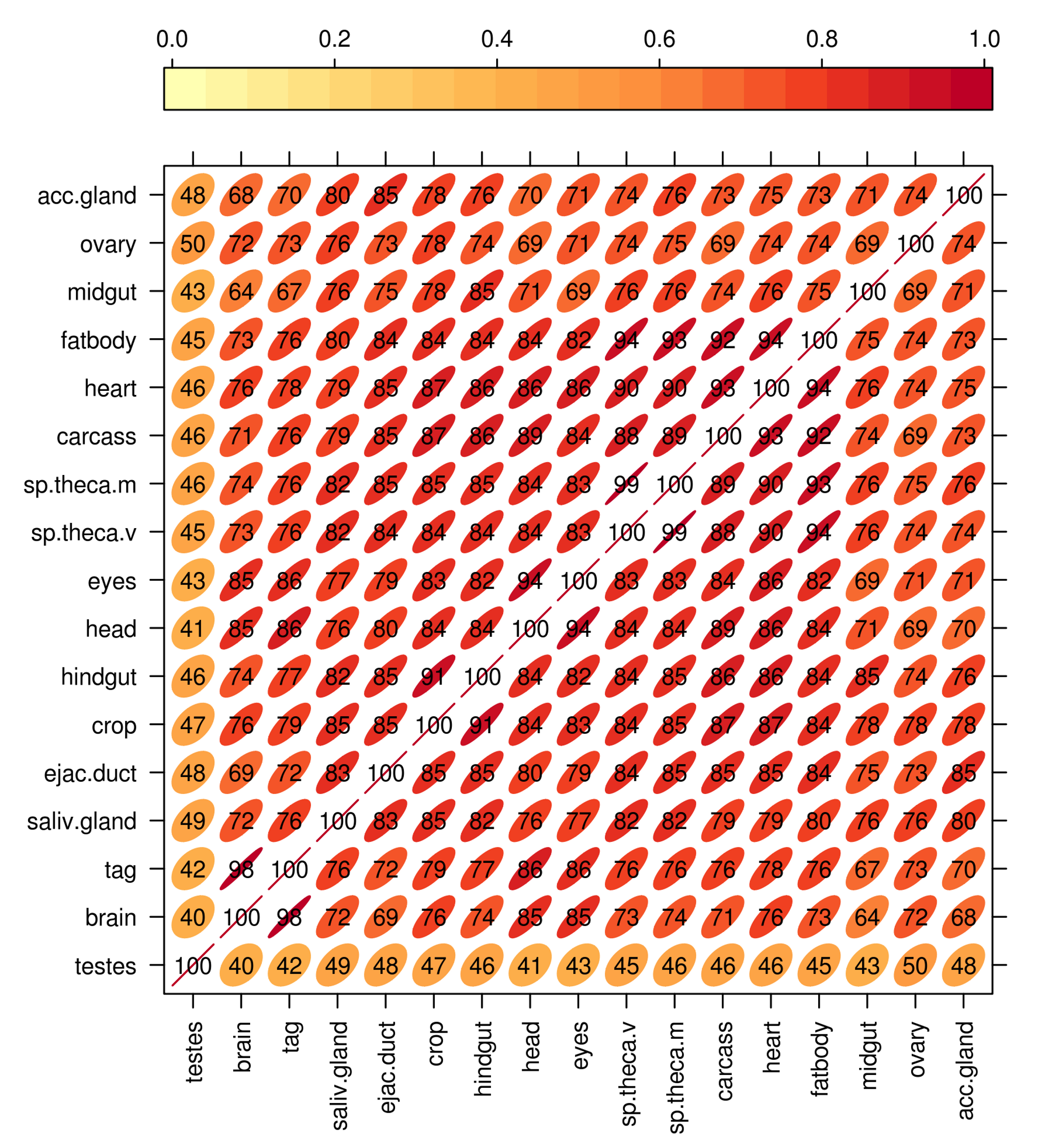

Supplement: Figure S3 — Correlation for gene expression in different tissues. Data from FlyAtlas [38]. (2.04 MB TIF) [file pbio.1000335.s003.tif]
